# Supplementary material for: Association between serum ferritin level and decreased diffusion capacity 3 months after the onset of COVID-19 pneumonia
Source: PLoS One. 2023 Feb 16;18(2):e0281249. doi: 10.1371/journal.pone.0281249 (PMC9934337; doi:10.1371/journal.pone.0281249)
Supplement: Attachment — Submitted filename: Response_to_Reviewers.doc [file pone.0281249.s001.doc]

October 13, 2022

Emily Chenette

Editor-in-Chief

*PLoS One*

Dear Editor:

We would like to thank you for your response and for giving us the opportunity to improve and resubmit our manuscript (ID:PONE-D-22-09029), entitled “Association between serum ferritin levels and decreased diffusion capacity 3 months after the onset of COVID-19 pneumonia.” We are hereby resubmitting a revised manuscript conforming to all of the reviewers’ comments. In particular, we have addressed all the reviewers’ comments in a point-by-point manner and revisions are indicated in blue (responses to Reviewer #1) and red (responses to Reviewer #2) font in the revised manuscript. We hope that the revised manuscript is now suitable for publication in your journal.

Thank you for your consideration. We look forward to hearing from you.

Sincerely,

Kyota Shinfuku, MD

Division of Respiratory Diseases, Department of Internal Medicine

The Jikei University Daisan Hospital

4-11-1 Izumihoncho Komae-shi, Tokyo 201-8601, Japan

Phone: ＋81-03-3480-1151

E-mail: [shinfuku.112@gmail.com](mailto:shinfuku.112@gmail.com)

**Reviewer 1**

The authors would like to thank the reviewer for their constructive critique to improve the manuscript. We have made every effort to address the issues raised and to respond to all comments. The revisions are indicated in blue font in the revised manuscript. Please, find next a detailed, point-by-point response to the reviewer's comments. We hope that our revisions will meet the reviewer’s expectations.

***・Small sample size.***

We appreciate the reviewer’s comment on this point. We agree with the reviewer that the sample size was small. However, we believe that it is important to note that the frequency of DLCO impairment was quite high. Based on the reviewer’s comments, we have discussed this issue as a limitation as follows:

“There are some limitations in this study. First, it was a retrospective study with a limited number of cases at a single institution.” (Lines 294–295)

***・Given the absence of baseline lung function measurement, it may be that 13/54 patients already had abnormal DLCO/ DLCO-Va prior to COVID-19.***

We appreciate the reviewer’s comment on this point. Based on the reviewer’s comments, we have discussed this issue as a limitation as follows:

“Second, there were no pulmonary function tests conducted before the onset of COVID-19. Cases of respiratory diseases associated with DLCO impairment, such as chronic obstructive pulmonary disease and interstitial pneumonia, were not included. Nevertheless, it could not be ascertained whether there was already reduced DLCO before the onset of COVID-19.” (Lines 295–299)

***・Abstract-background should define what’s known in the field or invoke interest to your question rather than describe your methods.***

We appreciate the reviewer’s comment on this point. Per the reviewer’s insightful suggestion, we have added more background information in the Abstract section as follows:

“The clinical factors associated with DLCO impairment, including blood biochemistry test parameters, remain unclear.” (Lines 23–24)

***・With a sample size of 13, it is difficult to make a conclusion that ferritin can be a long term lung function abnormalities in COVID-19. At best, you can report an association.***

We appreciate the reviewer’s comment on this point. Based on the reviewer’s suggestion, we have revised the conclusion in the Abstract and in the main text as follows:

“Decreased DLCO was the most common respiratory function impairment, and ferritin level was a significantly associated clinical factor. Serum ferritin level could be used as a predictor of DLCO impairment in cases of COVID-19 pneumonia.” (Lines 39–41)

“DLCO impairment was the most frequent disorder of respiratory function, and the peak serum ferritin level was associated with DLCO impairment. Ferritin level of >686.5 ng/mL could be a risk factor for DLCO impairment and indirectly reflects tissue damage with cytokine storms.” (Lines 302–305)

***・The manuscript presents no novel findings. How does this paper add to the scientific literature?***

We appreciate the reviewer’s comment on this point. It is known that DLCO impairment is common in COVID-19 pneumonia and that ferritin is associated with severity and mortality. However, to our knowledge, this is the first study to show a direct association between the peak ferritin level and DLCO impairment. We believe that this finding is novel and would contribute to literature. We have highlighted the novelty of the study as follows:

“To the best of our knowledge, no other blood biochemical markers associated with respiratory dysfunction have been reported to date. Moreover, the serum ferritin level has already been shown to be associated with COVID-19 severity and mortality [24]. However, to the best of our knowledge, no studies to date have shown a direct association between the elevated ferritin level and respiratory dysfunction. This study is the first to suggest a direct association of ferritin level and DLCO impairment with COVID-19 pneumonia.” (Lines 277–283)

“It is also possible that elevated ferritin levels could be used as a predictor of respiratory dysfunction due to viral pneumonia.” (Lines 291–293)

**・Please better clarify CT shadows in introduction. What is CT shadows?**

We appreciate the reviewer’s comment on this point. Based on the reviewer’s comments, we have provided a description in the Introduction section as follows:

“Severity of COVID-19 and the spread of abnormal chest shadows on computed tomography (CT) scans in COVID-19 pneumonia cases have been reported to be associated with DLCO reduction [12-14].” (Lines 67–69)

***・Standard definition of severe and critical COVID-19 has not been used.***

We appreciate the reviewer’s comment on this point. As the reviewer pointed out, we have revised the severity classification according to the NIH criteria. Based on the reviewer’s comments, we have corrected the description of the study design as follows:

“The severity was defined according to the COVID-19 treatment guidelines of the National Institute of Health [15]. In this study, patients were classified into three groups according to the aforementioned guidelines. Groups 1, 2, and 3 included patients who were hospitalized but did not require oxygen supplementation; those who were hospitalized and required conventional oxygen; and those who were hospitalized and required high-flow nasal cannula, noninvasive ventilation, invasive mechanical ventilation, or extracorporeal membrane oxygenation, respectively.” (Lines 80–87)

Moreover, we have corrected the description in Table 1.

***・How did you decide the blood test results selected? Was it just the data that was available?***

We would like to thank the reviewer for the questions. To respond to the reviewer’s question, we have added the following part to the revised manuscript:

“Blood tests were performed on admission and periodically thereafter throughout hospitalization. Then, we evaluated the blood sample data during the hospitalization period that may be related to the severity of COVID-19.” (Lines 92–94)

Moreover, we have provided more information concerning the frequency of blood test (days apart) in Table 1.

***・If you used CT findings to define your outcomes, mention this in the abstract.***

Per the reviewer’s insightful suggestion, we have added the following part to the Abstract:

“Clinical factors, including blood test parameters and abnormal chest shadows on computed tomography, of COVID-19 pneumonia associated with DLCO impairment were investigated.” (Lines 27–29)

***・Alternative causes of DLCO reduction were not ruled out.***

According to the reviewer’s insightful suggestion, we have added the following part to the Discussion section:

“Second, there were no pulmonary function tests conducted before the onset of COVID-19. Cases of respiratory diseases associated with DLCO impairment, such as chronic obstructive pulmonary disease and interstitial pneumonia, were not included. Nevertheless, it could not be ascertained whether there was already reduced DLCO before the onset of COVID-19.” (Lines 295–299)

***・Present findings in table 2. Table 1 should be demographics.***

We appreciate the reviewer’s comment on this point. Please note that we have made corrections in Tables 1 and 2. The revised parts are presented in blue font.

***・Please correct typing errors.***

We would like to apologize to the reviewer for the typing errors. Please note that we have double-checked and corrected the typing in the text and tables.

**Reviewer 2**

The authors would like to thank the reviewer for their constructive critique to improve the manuscript. We have made every effort to address the issues raised and to respond to all comments. The revisions are indicated in red font in the revised manuscript. Please, find next a detailed, point-by-point response to the reviewer's comments. We hope that our revisions will meet the reviewer’s expectations.

***1) Could not understand how AST, LDH and TSS that were significant in univariate analysis, did not show significance in multivariate analysis? Can explain the reason for this as Table 3 and Table 4 seem to show significant p-values for them for AUC and OR respectively. Is this related to the number of patients?***

We appreciate the reviewer’s comment on this point. As the reviewer indicated, the small sample size may have had an impact. Moreover, in the present study, multivariate analysis was performed for significant items in univariate terms, with cutoff values set using the ROC curves. The multivariate analysis used a stepwise approach, but only ferritin, which is most associated with DLCO impairment, remained as a variable. AST, LDH, and TSS, which were significant in the ROC curve AUC and univariate analyses, were excluded and did not remain. We thought that AST and LDH would not be significant in the multivariate analysis because of confounding factors, such as liver injury and muscle destruction. We also considered that TSS was removed and did not remain as a final factor because of the stronger association of ferritin with DLCO impairment.

***2) The relationship between DLCO and Ferritin is well documented in conditions such as Thalassemia especially when transfusion is needed. Were there any patients who were transfused? Also were there any underlying inflammatory diseases in the patients such as arthritis, autoimmune conditions that could have elevated the ferritin level?***

We would like to thank the reviewer for the comment. We again checked the transfusion history of the patients and comorbidities that may have contributed to the elevated ferritin level, such as autoimmune diseases, but none of the cases were applicable. Please note that we have discussed this issue in the revised manuscript as follows: “Apart from COVID-19, there were no cases in this study with a history of autoimmune disease or blood transfusions that contributed to elevated ferritin levels.” (Lines 274–276)
