# Supplementary material for: Association between serum ferritin level and decreased diffusion capacity 3 months after the onset of COVID-19 pneumonia
Source: PLoS One. 2023 Feb 16;18(2):e0281249. doi: 10.1371/journal.pone.0281249 (PMC9934337; doi:10.1371/journal.pone.0281249)
Supplement: Attachment — Submitted filename: Response_to_Reviewers.doc [file pone.0281249.s002.doc]

December 10, 2022

Emily Chenette

Editor-in-Chief

*PLoS One*

Dear Editor:

We would like to thank you for your response and for giving us the opportunity to improve and resubmit our manuscript (ID: PONE-D-22-09029), entitled “Association between serum ferritin levels and decreased diffusion capacity 3 months after the onset of COVID-19 pneumonia.” We are hereby resubmitting a revised manuscript conforming to all of the editor’s and reviewers’ comments. In particular, we have addressed all the reviewers’ comments in a point-by-point manner and revisions are indicated in blue (responses to the Editor) and red (responses to Reviewer #3) font in the revised manuscript.

Revisions that are common to the editor's and reviewer's comments are indicated in green font. We hope that the revised manuscript is now suitable for publication in your journal.

Thank you for your consideration. We look forward to hearing from you.

Sincerely,

Kyota Shinfuku, MD

Division of Respiratory Diseases, Department of Internal Medicine

The Jikei University Daisan Hospital

4-11-1 Izumihoncho Komae-shi, Tokyo 201-8601, Japan

Phone: ＋81-03-3480-1151

E-mail: [shinfuku.112@gmail.com](mailto:shinfuku.112@gmail.com)

**Academic editor**

The authors would like to thank the editor for their constructive critique to improve the manuscript. We have made every effort to address the issues raised and to respond to all comments. The revisions are indicated in blue font in the revised manuscript. Revisions that are common to the corrections to the reviewer's remarks are noted in green font. Please, find next a detailed, point-by-point response to the editor's comments. We hope that our revisions will meet the reviewer’s expectations.

***・****In particular, taking into account the suggestions of reviewer #3, I would ask you to improve the methodological precision described in the M&M session, particularly in the first part where you describe the groups, explaining well the whole methodology of the construct of your paper.*

We appreciate the reviewer’s comment on this point. We added a description of the research methods in the study design section as follows: “Enrolled patients were those who were hospitalized with COVID-19 pneumonia during the period and who requested outpatient follow-up after the sequelae symptoms and respiratory dysfunction were explained to them. From the standpoint of infection control, an outpatient examination and CT scan were performed 2 months later and it was confirmed that the patient was improving. Then, pulmonary function tests were performed 3 months later.” (Lines 81-86)

We then added the time to the examination for each group in Tables 1 and 2.

*Again, personally I have two more considerations. First, as you also suggested in the Discussion, it is possible that the differences you found with respect to the Italian literature are also related to racial differences; however, nowhere do I find an indication of the percentage of the Asian, Caucasian, black population that you analysed. This data needs to be implemented.*

We appreciate the reviewer’s comment on this point. We have added a description of the race of the patients in Table 1. In addition, based on the editor’s comments, we have corrected the description of the discussion section as follows: “Then, in our study, all patients were Asian, whereas the Italian study did not provide details but may have included more whites. Racial differences could also contribute to the differences in the proportion of residual symptoms between studies.” (Line 264-267)

*Secondly, it is also possible that the difference between the Italian study and yours is linked to a different pharmacological treatment (steroids, anticoagulants, anti-IL6, etc.) which had not yet been implemented at the time of the first wave. This consideration must also be implemented too.*

We appreciate the reviewer’s comment on this point. We have added a description in the discussion section as follows: “In addition, the Italian study was from the first wave of the infection, when the efficacy of dexamethasone and antiviral drugs had not been established. Differences in treatment may also have affected residual symptoms.” (Lines 267-269)

**Reviewer 3**

The authors would like to thank the reviewer for their constructive critique to improve the manuscript. We have made every effort to address the issues raised and to respond to all comments. The revisions are indicated in red font in the revised manuscript. Revisions that are common to the corrections to the editor's remarks are noted in green font. Please, find next a detailed, point-by-point response to the reviewer's comments. We hope that our revisions will meet the reviewer’s expectations.

*Overall, I agree with prior reviews in general. This retrospective review of a single institution’s experience reveals that peak serum ferritin measures at the time of admission are the most predictive of a reduced DLCO and DLCO/VA at 3 months. I need to reiterate that the number of subjects is small, and the general lack of clinical characterization of the cohort make the results less than convincing.*

*And there is no description of how these subjects were enrolled. The authors state that the CT was done 2 months after onset, and PFTs were done 3 months after onset. Why? And how can you be so sure that the CTs were done exactly 2 months after and the PFTs done exactly 3 months after? There is no mention of a protocol.*

We appreciate the reviewer’s comment on this point. We added a description of how the eligible patients were enrolled and how the CT scan was performed after 2 months and the pulmonary function test after 3 months in the study design section as follows: “Enrolled patients were those who were hospitalized with COVID-19 pneumonia during the period and who requested outpatient follow-up after the sequelae symptoms and respiratory dysfunction were explained to them. From the standpoint of infection control, an outpatient examination and CT scan were performed 2 months later and it was confirmed that the patient was improving. Then, pulmonary function tests were performed 3 months later.” (Lines 81-86)

We then added the time to the examination for each group in Tables 1 and 2.

*There is no discussion of why ferritin levels would correlate with a reduction in DLCO. What mechanism of action would that be?*

We appreciate the reviewer’s comment on this point. We added a description of the association between ferritin levels and DLCO reduction in the discussion section as follows:

“The uncontrolled virus invades the tissues and infects cells in the alveolar epithelium, airway epithelium, and pulmonary capillary endothelium, causing inflammatory cell death and leading to respiratory dysfunction. To cope with the dead inflammatory cells, an even larger number of immune cells infiltrate into the lungs, presumably resulting in excessive production of inflammatory cytokines [27]. Ferritin is presumed to indirectly reflect this cytokine storm. The association between DLCO impairment and ferritin level suggests that elevated ferritin may indicate inflammatory cell death associated with respiratory dysfunction.” (Lines 299-306)

*The authors are encouraged to develop a prediction model, using multiple clinical, lab, and radiologic characteristics that could be validated prospectively. But as it stands now, I’m not convinced this adds much to the existing literature regarding reduced DLCO after Covid.*

We appreciate the reviewer’s comment on this point. We added a corresponding description in the discussion section as follows: “First, it was a retrospective study with a limited number of cases at a single institution. The applicants were included and selection bias is taken into account. A larger prospective study of clinical factors predicting respiratory dysfunction after COVID-19 is needed.” (Line 311-314)
